# Decreased CD44v3 expression impairs endometrial stromal cell proliferation and decidualization in women with recurrent implantation failure

**DOI:** 10.1186/s12958-022-01042-w

**Published:** 2022-12-16

**Authors:** Xiaowei Zhou, Yi Cao, Mingjuan Zhou, Mi Han, Mengyu Liu, Yanqin Hu, Bufang Xu, Aijun Zhang

**Affiliations:** 1 Department of Obstetrics and Gynecology, Ruijin Hospital, Shanghai Jiao Tong University School of Medicine, 197 Ruijin 2Nd Road, Shanghai, 200025 China; 2 Department of Obstetrics and Gynecology, Minhang Hospital, Fudan University, 170 Xin Song Road, Shanghai, 201100 People’s Republic of China; 3 Department of Histo-Embryology, Genetics and Developmental Biology, Shanghai Key Laboratory of Reproductive Medicine, Shanghai Jiao Tong University School of Medicine, 280 South Chongqing Road, Shanghai, 200025 China

**Keywords:** Recurrent implantation failure, Endometrial receptivity, CD44v3, Proliferation, Endometrial decidualization

## Abstract

**Background:**

The precise pathogenesis of poor endometrial receptivity in recurrent implantation failure (RIF) remains unclear. This study was aimed at exploring the effects of different CD44 isoforms in the mid-secretory phase endometrium on endometrial receptivity in women with RIF.

**Methods:**

Mid-secretory phase endometrial tissue samples were obtained from the following two groups of women who had undergone IVF: (a) 24 patients with RIF and (b) 18 patients with infertility due to tubal obstruction, who had achieved a successful clinical pregnancy after the first embryo transfer in IVF (control group). Identification of differentially expressed CD44 isoforms in endometrial tissues was assessed using immunohistochemistry, qPCR, and western blotting. Effects of overexpression and knockdown of CD44v3 on proliferation and decidualization of immortalized human endometrial stromal cells (T-HESCs) and primary HESCs were investigated by qPCR and western blot analysis. A heterologous coculture system of embryo implantation was constructed to mimic the process of trophoblast invasion during implantation.

**Results:**

The expression of CD44v3 was significantly higher in the mid-secretory phase of endometrial stromal cells than in the proliferation phase, but was notably lower in RIF patients. Knockdown of CD44v3 significantly downregulated cell proliferation both in T-HESCs and HESCs. The expression of decidualization markers, prolactin (PRL) and insulin like growth factor binding protein-1 (IGFBP1), was notably decreased following the knockdown of CD44v3, whereas the expression of both PRL and IGFBP1 increased after its overexpression in HESCs. Furthermore, the CD44v3-knockdown HESCs displayed significant deficiency in supporting trophoblast outgrowth in a coculture system of embryo implantation; however, overexpression of CD44v3 in HESCs promoted trophoblast outgrowth.

**Conclusion:**

The reduced expression of CD44v3 suppresses the proliferation and decidualization of HESCs, which might play a pivotal role in poor endometrial receptivity in women with RIF.

**Supplementary Information:**

The online version contains supplementary material available at 10.1186/s12958-022-01042-w.

## Background

Reproductive failure is a major social and economic problem. In recent years, breakthroughs in assisted reproductive technology have improved outcomes for couples who previously failed to conceive and achieve viable pregnancy [1]. Nevertheless, a new challenge has emerged, as approximately 10% of infertile women who undergo in vitro fertilization and embryo transfer (IVF-ET) cycles suffer from recurrent implantation failure (RIF) [2]. RIF refers to an unsuccessful clinical pregnancy after a minimum of three transfers of at least four morphologically good quality embryos (grade above 3BB or score ≥ 7) into a normal uterus [3]. Disturbed immunological factors and maternal endocrine abnormalities, particularly inadequate endometrial receptivity, constitute some of the RIF etiologies [4]. However, the precise pathogenesis of poor endometrial receptivity in RIF remains unclear.

During the secretory phase, the endometrium undergoes decidualization, and endometrial stromal cells proliferate and differentiate into decidual cells to attain endometrial receptivity [5]. Decidualization, which plays key roles in embryo support, nutritional furnishing, endocrine regulation, and immune modulation, is a prerequisite for embryo implantation in some mammals, including mice and humans [6]. For example, loss of BMP2 in the uterus renders mice infertile, causing failure of decidualization. Additionally, uterine Fst-cKO mice show severe fertility defects, including a poor decidualization response, with relatively low levels of stromal proliferation and differentiation [7]. In a previous study, we demonstrated that reduced expression of PIBF1 in the mid-secretory phase of RIF patients inhibits the proliferation and decidualization of human endometrial stromal cells (HESCs) [8].

The transmembrane glycoprotein, CD44, is thought to participate in various cellular processes, including regulation of cell proliferation, migration, and adhesion [9]. Therefore, it may be necessary for the maintenance of pregnancy. For instance, the CD44-mediated inchoate attachment between endometrial epithelial cells and trophectoderm and relatively low CD44 expression in decidual cells were found to be associated with unexplained incidences of miscarriage [10, 11]. Furthermore, it has been reported that CD44 is not expressed in human endometrial cells during the proliferative phase, whereas it shows intense expression in the mid and late secretory phases [12, 13]. However, because CD44 frequently shows alternative spliced variants, including the shortest standard form of CD44 (CD44s) and multiple CD44 variants (CD44v), the unique isoforms of CD44 expressed in the endometrium during the menstrual cycle have not been fully elucidated. Thus, whether CD44 isoforms participate in regulating the development of endometrial receptivity in RIF is unclear. In this study, we explored the effects of different CD44 isoforms, particularly CD44v3, in the mid-secretory phase on endometrial receptivity in women with RIF.

## Methods

### Patients

Studies of human subjects were approved by the Institutional Ethics of Ruijin Hospital, School of Medicine, Shanghai Jiao Tong University (2012–57). Participants were enrolled for 18 months from Dec 2019 to May 2021 at the Reproductive Medical Center of Ruijin Hospital. We recruited 24 RIF patients aged between 25 and 35 years who had experienced three or more previous failed cycles wherein at least four good quality embryos were transferred. The comparison group included 18 women with infertility due to tubal obstruction, who had achieved a successful clinical pregnancy after the first embryo transfer in IVF. All patients in the comparison group underwent cleavage-stage embryo transfer, and blastocyst was transferred in patients after being diagnosed with RIF. The exclusion criteria were as described previously [14]. Briefly, individuals with uterus pathology, hydrosalpinx, adenomyosis, polycystic ovary syndrome, autoimmune disease, endometriosis, and chromosome abnormalities were excluded. Mid-secretory phase endometrial samples were obtained at day LH + 7 through pipe suction curettage (LILYCLEANER; Shanghai Jiabao Medical Healthcare Science and Technology Ltd., China).

Furthermore, 12 women in the early proliferative (days 5–7 of the cycle) and 4 women in the late proliferative (days 11–14) matching the same criteria as the control group were enrolled.

### Cell culture

Human telomerase reverse transcriptase-immortalized human endometrial stromal cells (T-HESCs; which are commonly used to study HESCs and endometrial receptivity) and Ishikawa cells (human endometrial carcinoma cell line, which are commonly used to study endometrial epithelial cells and embryo adhesion), were acquired from the European Collection of Authenticated Cell Cultures (ECACC; Salisbury, UK). Primary human endometrial epithelial cells (HEECs) and HESCs were isolated as previously described [8]. Briefly, endometrial samples were minced and digested for 30 min using 1 mg/mL collagenase type I (Thermo Fisher Scientific, Waltham, MA, USA) at 37 °C. The mixture was then successively passed through 100 and 40 µm sieves (Millipore Sigma, Burlington, MA, USA), and the flushing and reverse flushing filtrates from the 40 µm sieve were centrifuged for 5 min at 100 × *g* to isolate HESCs and HEECs. T-HESCs, Ishikawa cells, primary HEECs, and HESCs were cultured in DMEM/F12 medium (Thermo Fisher Scientific), supplemented with 10% (*v/v*) fetal bovine serum (FBS), 100 IU/mL penicillin, and 100 μg/mL streptomycin (Thermo Fisher Scientific), at 37 °C according to standard procedures and harvested using 0.25% (*w*/*v*) trypsin–EDTA (Thermo Fisher Scientific).

### RNA isolation and RT-PCR analysis

Total RNA was extracted from the samples according to the manufacturer’s instructions (Takara Biomedical Technology (Beijing) Co., Ltd., Beijing, China) and reverse transcribed using PrimeScript™ RT Master Mix (Takara Biomedical Technology (Beijing) Co., Ltd.). Reverse transcription-quantitative PCR was performed using SYBR Green Master Mix (Takara Biomedical Technology (Beijing) Co., Ltd.) and Applied Biosystems 7500 Real time PCR System (Applied Biosystems, Waltham, MA, USA). The sequences of gene-specific primers are listed in Supplemental Table 1. Relative quantification of mRNA levels was performed using the comparative cycle threshold (Ct) method, with *GAPDH* as the reference gene. All tests were repeated at least three times.

### Protein isolation and western blot analysis

Proteins were extracted by lysing endometrial samples and cells with RIPA lysis buffer (Thermo Fisher Scientific), supplemented with a protease inhibitor cocktail (Roche, Basel, Switzerland). The lysates were then centrifuged at 12,000 × *g* for 10 min at 4 °C, and the supernatant was collected. Samples with 30 μg proteins were separated via 10% SDS-PAGE, and the resolved proteins were transferred onto PVDF membranes (Millipore Sigma) that were blocked with 5% non-fat milk in TBST for 1 h. The membranes were then incubated overnight at 4 °C with protein-specific primary antibodies. Following washing and incubation with a corresponding HRP-conjugated antibody at room temperature for 1 h, the bands were visualized via enhanced chemiluminescence (Millipore Sigma). Primary antibodies against CD44v3 (1 μg/mL; R&D Systems, Minneapolis, MN, USA) and GAPDH (1:1,000; Cell Signaling Technology, Danvers, MA, USA) were used in this study. Quantification was performed using the ImageJ software and normalized to GAPDH levels.

### Immunohistochemical staining

Tissue specimens were fixed with 4% formalin. Paraffin Sects. (5 μm) were prepared and fixed. Antigen retrieval was performed by incubating the cells in buffered citrate for 15 min at 105 °C. The sections were blocked with 5% *w*/*v* bovine serum albumin for 30 min and then incubated with primary antibodies against CD44s (1:100; Abcam, Cambridge, UK), CD44v3 (8 μg/mL; R&D Systems), and CD44v6 (1:100; Abcam) overnight at 4 °C. The slides were then stained with horseradish peroxidase-conjugated secondary antibodies, followed by counterstaining with diaminobenzidine (Agilent Technologies, Santa Clara, CA, USA) and hematoxylin. Images were visualized using a microscope (Olympus Corporation, Tokyo, Japan) or captured using a panoramic scanner PANNORAMIC (3DHISTECH Ltd., Budapest, Hungary). Each specimen was assigned a score according to the intensity of staining (negative staining = 0; week staining = 1; moderate staining = 2, and strong staining = 3). To diminish the manual errors, two pathologists performed the scoring independently. CD44s, CD44v3, and CD44v6 were quantified using the formula: IHC score = Σ (% of immunostained cells × intensity of staining).

### SiRNA-mediated knockdown and plasmid overexpression studies

The following siRNAs were purchased from GenePharma (Shanghai, China): Control siRNA (sense: 5′-UUCUCCGAACGUGUCACGUTT-3′, antisense: 5′- ACGUGACACGUUCGGAGAATT-3′), CD44v3 siRNA1 (sense: 5′- AGGCAUUGAUGAUGAUGAAUU-3′, anti-sense: 5′-UUCAUCAUCAUCAAUGCCUUU-3′), and CD44v3 siRNA2 siRNA (sense: 5′-UGAAGAUGAAAGAGACAGAUU-3′, anti-sense: 5′-UCUGUCUCUUUCAUCUUCAUU-3′), CD44v3 siRNA3 siRNA (sense: 5′-GGCUUUCAAUAGCACCUUGUU-3′, anti-sense: 5′-CAAGGUGCUAUUGAAAGCCUU-3′). Mock and CD44v3 overexpression plasmids were purchased from FulenGen (Guangzhou, China). Cell transfection was conducted using the X-tremeGENE 9 DNA transfection reagent (Roche). Cells were transfected with shRNAs or plasmids using standard procedures.

### Proliferation assay

Cell proliferation was examined using the Cell Counting Kit-8 (CCK-8; Dojindo China Co., Ltd., Shanghai, China). Briefly, cells were seeded in 96-well plates at a density of 1,000 cells per well. A total of 10 μL CCK-8 solution was added to each well, and the optical density was measured at 450 nm using a microplate reader. The experiment was repeated three times.

### In vitro decidualization activity assay

T-HESCs and HESCs separated from the late proliferative phase of the endometrium of the control group, were transfected with CD44v3 siRNA or CD44v3 overexpression plasmid. After 48 h transfection, the cells were cultured in a serum-free DMEM-F12 medium with 10 nM *β*-estradiol (Sigma Aldrich), 1 μM progesterone (Sigma Aldrich), and 1 mM 8-Br-cAMP (Abcam) for 72 h. Total RNA and protein were extracted, and the expression of the decidual markers, prolactin (PRL) and insulin like growth factor binding protein-1 (IGFBP1), was evaluated via RT-qPCR and western blot analysis.

### Migration assay

T-HESCs, HESCs and Ishikawa cells (1 × 10^6^ cells), with or without treatment, were seeded in 6-well plates to reach subconfluence overnight. The monolayer of cells was then scratched with a pipette tip to create a cell-free wound. The plates were washed two times with PBS and further culturing was done in fresh serum-free medium. Wound healing ability was quantified by measuring the percentage closure of the wound at 0, 24, and 48 h. Three independent experiments were conducted.

### Embryo outgrowth analysis

Trophoblast outgrowth analysis was performed as described [3]. In brief, HESCs were isolated from the late proliferative phase endometrium from the control group and cultured in a 24-well plate. After 48 h, cells transfected with CD44v3 siRNA or CD44v3 overexpression plasmids were decidualized as described above. Hatched mice blastocysts with normal morphology were then cocultured with confluent monolayers of decidualized HESCs in DMEM/F12 complete medium. The trophoblast outgrowth areas were outlined and calculated using Image J 1.46r.

### Statistical analysis

Data are presented as mean ± SEM and were analyzed using the SPSS software (version 23.0; SPSS Inc., Chicago, IL, USA). For continuous variables, statistical analysis between two groups was performed using the two tailed Student’s *t*-test when data met the normal distribution criterion. For more than two groups, statistical analysis was performed using one way analysis of variance (ANOVA) with the Bonferroni test for establishing the normality and homogeneity of variance data. In addition, the nonparametric test of Mann–Whitney *U* test was applied for non-normal data and the data are represented as median and range. The receiver operating characteristic (ROC) curve was analyzed using the SPSS software. Statistical significance was set at *p* < 0.05.

## Results

### Demographics

Demographic details of the women recruited in the study are summarized in Table 1. Except for the number of embryos transferred, other indices were not significantly different between the control and RIF groups (*p* > 0.05).

**Table 1:** Demographic characteristics of women recruited in this study

	Control (n = 18)	RIF (n = 24)	*P*
Age (y)	30.78 ± 3.54	31.75 ± 3.10	0.349
BMI (kg/m^2^)	22.40 ± 3.52	21.87 ± 1.98	0.544
Basal FSH (mIU/mL)	6.28 ± 1.55	6.62 ± 1.85	0.541
Basal LH (mIU/mL)	4.20 ± 2.41	3.23 ± 1.54	0.119
Basal E2 (pg/mL)	37.08 ± 13.31	41.25 ± 18.48	0.422
Endometrial thickness on the day of embryo transfer (mm)	8.59 ± 0.38	8.43 ± 0.39	0.180
Times of embryo transfer executed	1(1, 1)	4(3, 10)	< 0.001
Number of embryos per transfer	1.61 ± 0.50	1.64 ± 0.48	0.803
Average score of transferred day-3 embryos	7.59 ± 0.63	7.66 ± 0.71	0.630
Score of transferred blastocysts	(0/0,0%)	3BB (21/78, 27%) 4AB (30/78, 38%) 4BB (27/78, 35%)	

Data are presented as mean ± SD. The difference between two groups was analyzed by an independent sample *t*-test, except for the number of embryo transfers calculated using the independent samples Mann–Whitney U test (median, range). BMI, body mass index; FSH, follicle stimulating hormone; LH, luteinizing hormone; E_2_, estradiol

### CD44v3 was decreased in endometrial stroma of RIF cases

**Fig. 1:**
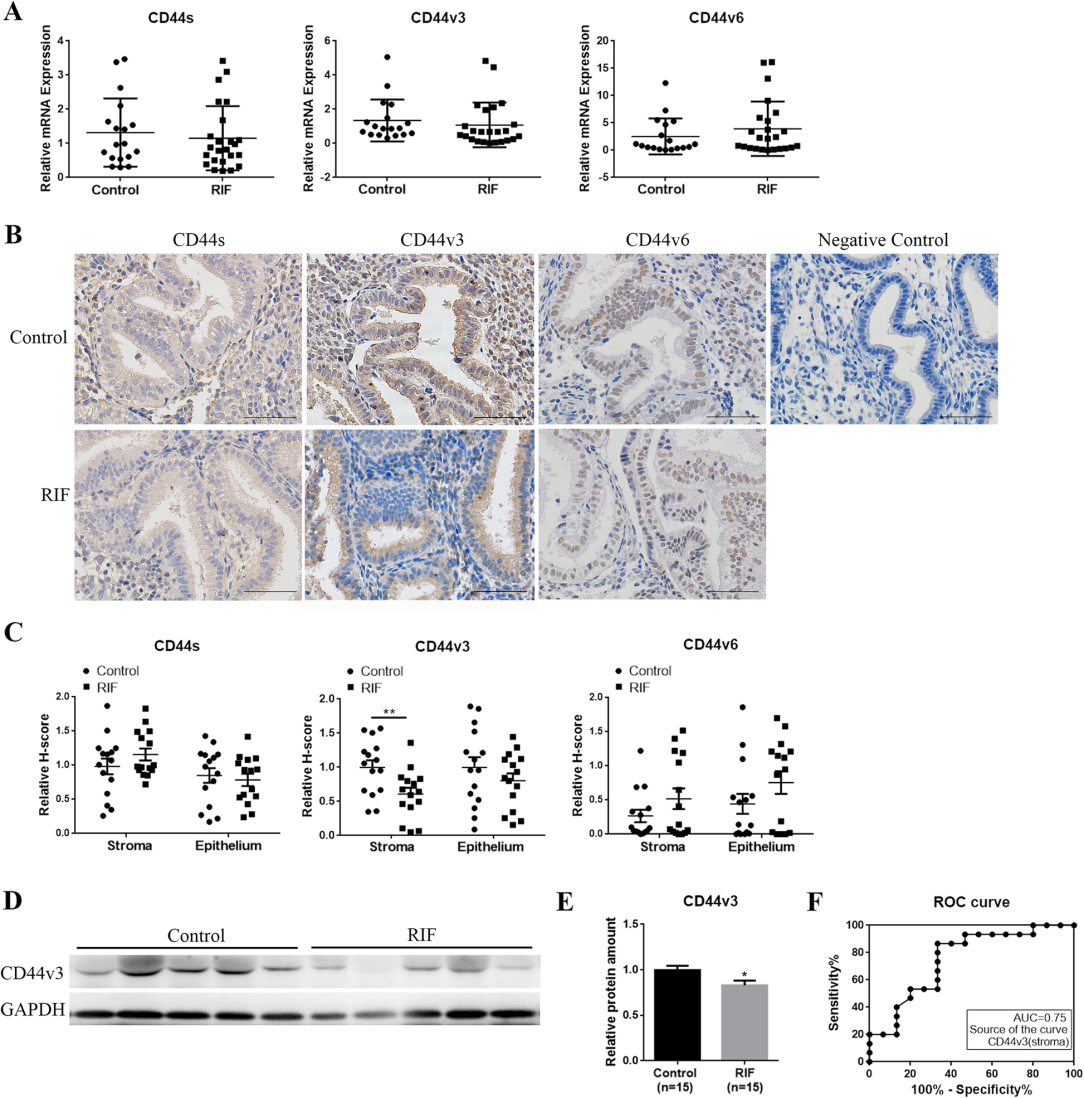
Expression of CD44v3 in the endometrium of women with RIF (**A**) mRNA levels of CD44s, CD44v3, and CD44v6 in the endometrium of women in the control (*n* = 18) and RIF (*n* = 24) groups. **B** Immunohistochemical staining and (**C**) semiquantitative analysis of CD44s, CD44v3, and CD44v6 in the control and RIF groups (*n* = 15 per group). **D** Representative western blots and (**E**) densitometric quantification of CD44v3 in the control and RIF groups. (F) ROC curve for determining the expression of CD44v3 in endometrial tissue. Bar = 50 µm. *p < 0.05; **p < 0.01

RT-qPCR analysis of the endometrial tissues from the mid-secretory phase revealed no difference in the mRNA levels of CD44s, CD44v3, and CD44v6 between the control (*n* = 18) and RIF groups (*n* = 24) (Fig. 1A, *p* < 0.05). Immunostaining was then performed to investigate the cellular localization and levels of CD44 isoforms in human endometrial tissue. The results showed that CD44s and CD44v3 were localized in the epithelial and stromal cells of the endometrium (Fig. 1B). CD44v6 showed three different expression patterns (Fig. 1B, Supplemental Fig. 1): 1. barely observed on both endometrial epithelium and stromal cells (7/15 in the control group; 5/15 in the RIF group); 2. mostly restricted to epithelial cells (3/15 in the control group; 3/15 in the RIF group); 3. located in both uterine epithelium and stroma (5/15 in the control group; 7/15 in the RIF group).

CD44s and CD44v6 showed similar expression in both the groups; however, the levels of CD44v3 were significantly reduced in the RIF group compared with those in the control group (Fig. 1B–E, *p* < 0.05), particularly in stromal cells (Fig. 1B, *p* < 0.01). To evaluate the clinical significance of this finding, an ROC curve was derived to measure the performance of a binary classifier. As shown in Fig. 1F, the ROC area under the curve was 0.75 for CD44v3 tissue measurements, indicating a potential functional role of CD44v3 in the diagnosis of endometrial receptivity.

### CD44v3 was upregulated in the stroma of mid-secretory phase endometrium and was induced by E2 and P4 treatments in HESCs

**Fig. 2:**
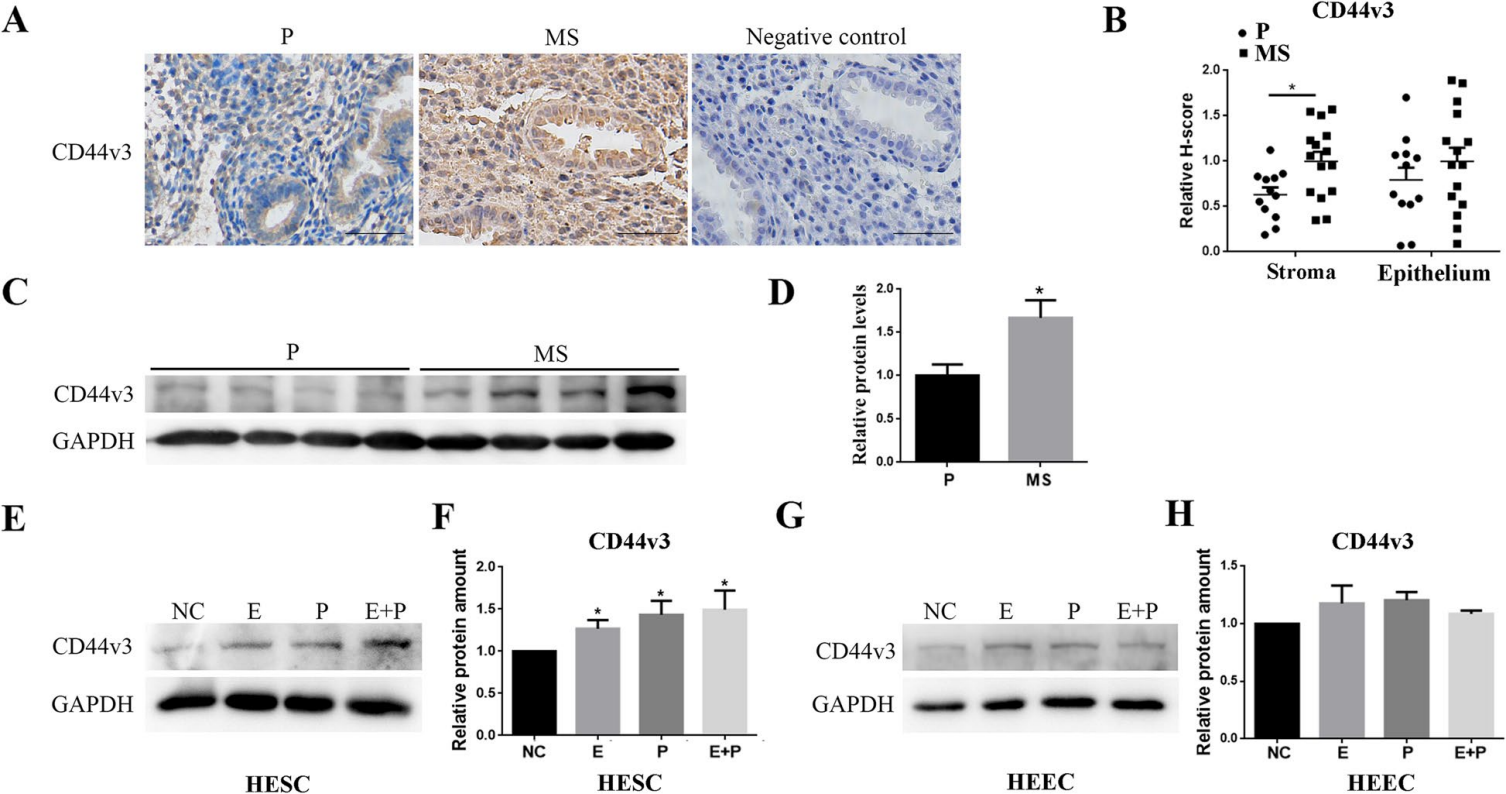
Expression of CD44v3 in endometrial tissues and primary endometrial cells after E_2_ and P_4_ treatments (A)Immunohistochemical staining and (B) semiquantitative analysis of CD44v3 during the proliferative (*n* = 12) and mid-secretory phases (*n* = 15) in the control patients. (C) Western blot and (D) statistical analyses of CD44v3 expression in the endometrium during the proliferative and mid-secretory phases in the control group. (E and G) Western blot and (F and H) statistical analyses of CD44v3 expression in primary HESCs and HEECs with or without estrogen (E_2_) and progesterone (P_4_) treatment for 72 h. P, proliferative phase; MS, mid-secretory phase. Bar = 50 µm. *p < 0.05.

As assessed from the results of immunostaining assay, the expression of CD44v3 was significantly higher during the mid-secretory phase (*n* = 12) than it was during the early proliferative phase of the endometrium (*n* = 15), especially in the endometrial stromal cells (Fig. 2A–D, *p* < 0.05). Furthermore, we found that when stimulated with E_2_ and/or P_4_, CD44v3 levels in primary endometrial stromal cells were significantly increased, with no significant change in primary endometrial epithelial cells (Fig. 2E–H, *p* < 0.05).

### Knockdown of CD44v3 suppressed the proliferation and decidualization of T-HESCs

**Fig. 3:**
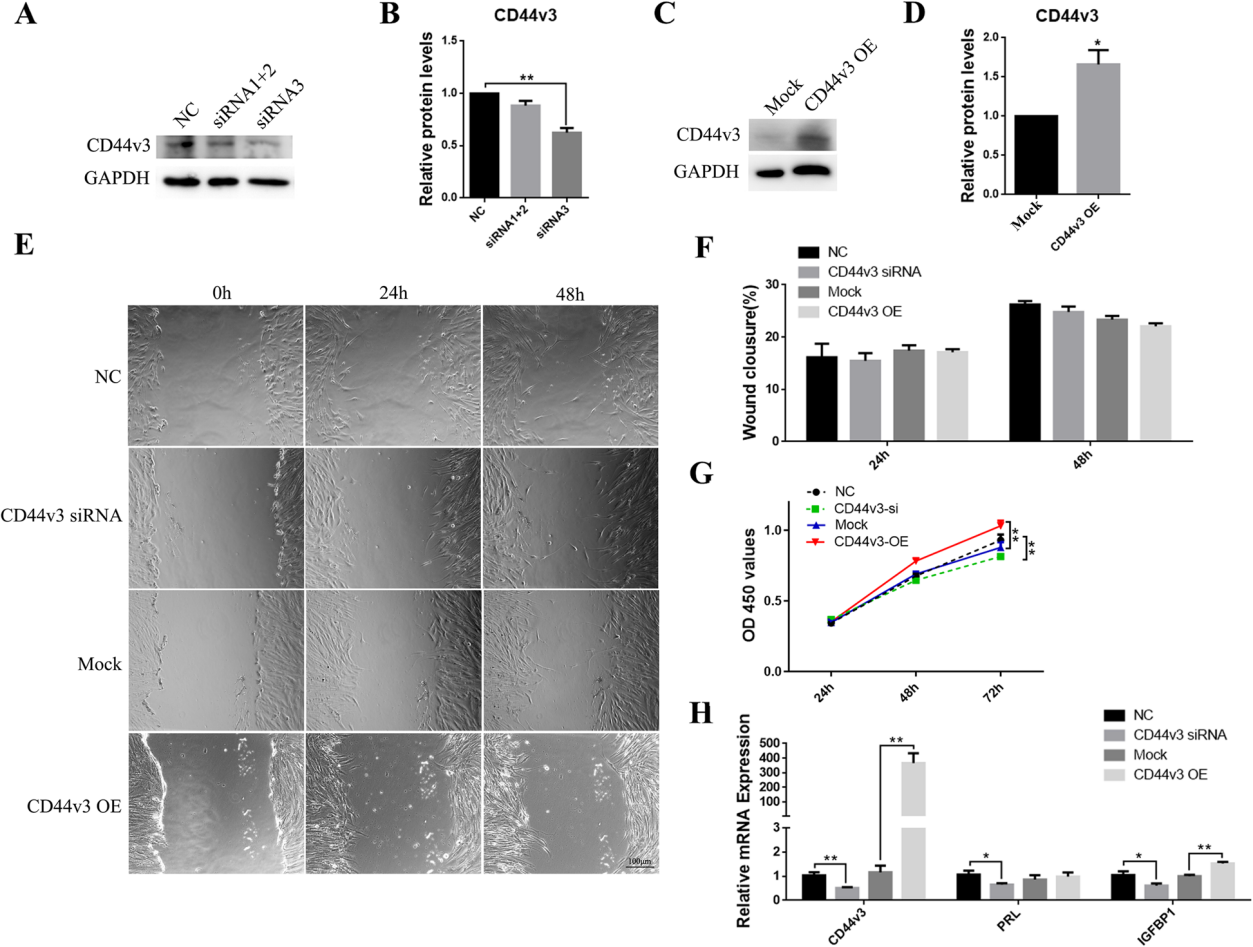
Changes in cell proliferation and stromal cell decidualization following CD44v3 knockdown/overexpression in T-HESCs (A, C) Western blot and (B, D) densitometric quantification of CD44v3 after the knockdown/overexpression of CD44v3 in T-HESCs. (E) Wound healing analysis and (F) semiquantitative analysis of wound closure in T-HESCs after the knockdown/overexpression of CD44v3. (G) Cell proliferation analyses after the knockdown/overexpression of CD44v3 in T-HESCs. (H) Expression levels of CD44v3, PRL, and IGFBP1 after the knockdown/overexpression of CD44v3 during the in vitro decidualization assay in T-HESCs. *p < 0.05; **p < 0.01

The effects of knockdown and overexpression of CD44v3 in T-HESCs were examined using western blot analysis (Fig. 3A–D, *p* < 0.01). The knockdown or overexpression of CD44v3 did not affect the rate of migration of T-HESCs (Fig. 3E–F). Notably, cell proliferation decreased following the knockdown of CD44v3 and increased following its overexpression (Fig. 3G, *p* < 0.05). To explore whether CD44v3 is involved in the decidualization pathways, an in vitro decidualization model of T-HESCs was constructed by treating the cells with E_2_, *P*_4_, and 8-bromo-cAMP. The results showed that mRNA expression levels of the two decidualization markers, PRL and IGFBP1, decreased following the knockdown of CD44v3 in T-HESCs. However, the levels of IGFBP1 increased after the overexpression of CD44v3 (Fig. 3H, *p* < 0.05).

### Knockdown of CD44v3 suppressed the proliferation and decidualization of HESCs

**Fig. 4:**
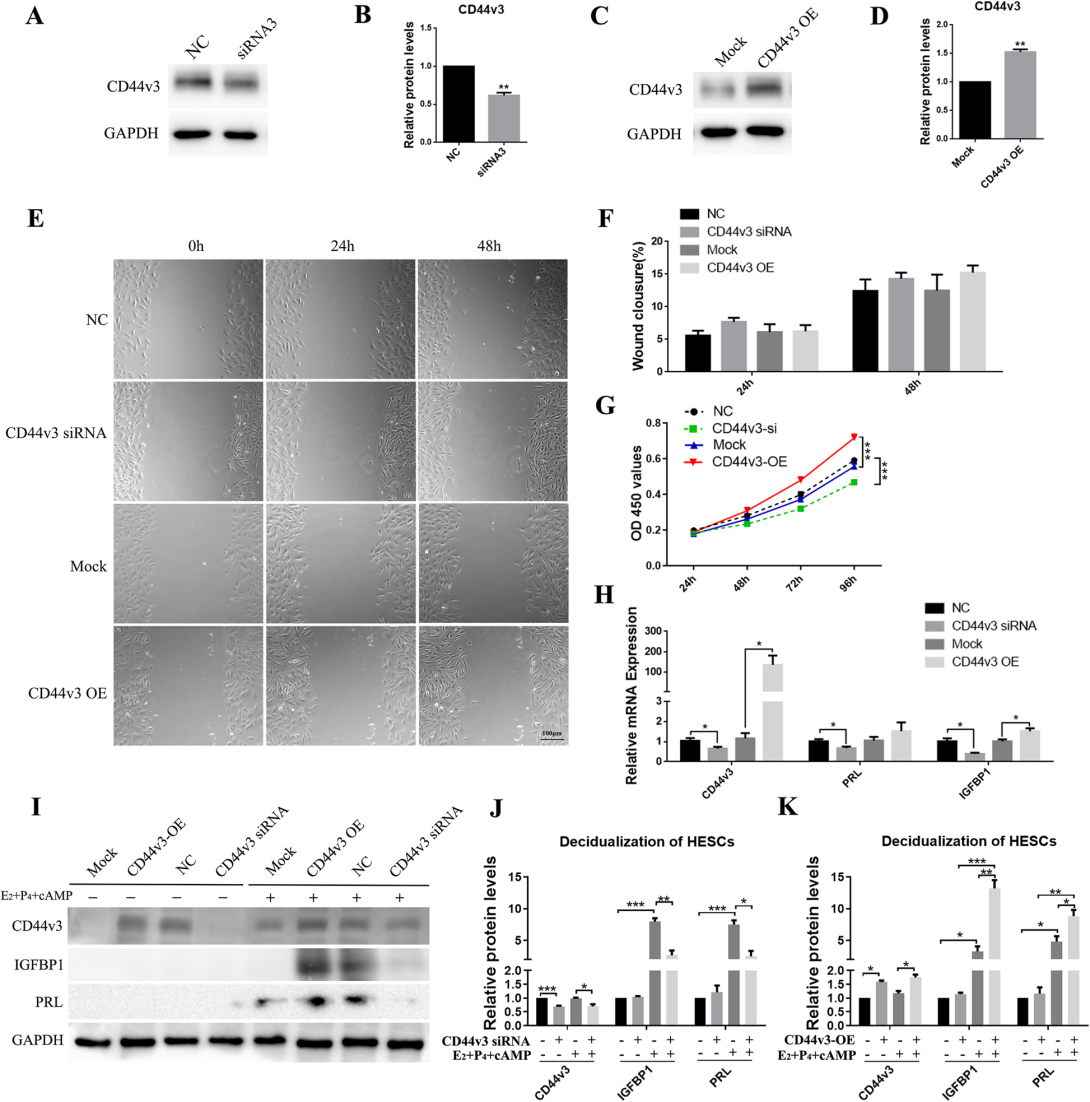
Changes in cell proliferation and stromal cell decidualization following CD44v3 knockdown/overexpression in primary HESCs (A, C) Western blot and (B, D) densitometric quantification of CD44v3 expression after the knockdown/overexpression of CD44v3 in primary HESCs. (E) Wound healing analysis and (F) semiquantitative analysis of wound closure in primary HESCs after the knockdown/overexpression of CD44v3. (G) Cell proliferation after the knockdown/overexpression of CD44v3 in primary HESCs. (H) mRNA expression of CD44v3, PRL, and IGFBP1 after the knockdown/overexpression of CD44v3 during the in vitro decidualization assay in primary HESCs. (I) Western blot and (J, K) densitometric quantification of CD44v3, PRL, and IGFBP1 after the knockdown/overexpression of CD44v3 during the in vitro decidualization assay in primary HESCs. *p < 0.05; **p < 0.01

**Fig. 5:**
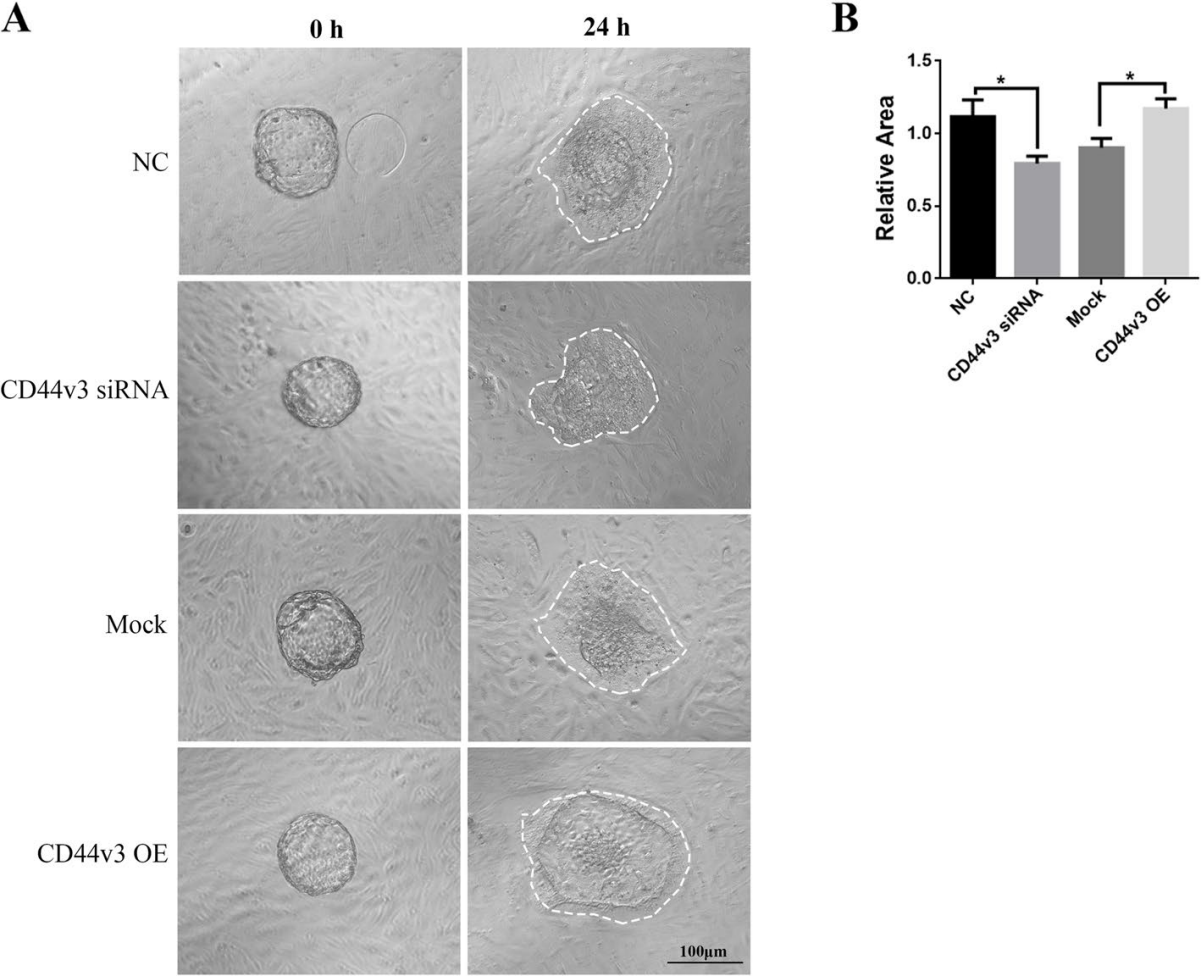
CD44v3 knockdown in ESCs inhibits trophoblast outgrowth **in vitro **(A) Representative images of spreading mouse trophoblast (white line) cocultured on negative control and CD44v3 knockdown HESCs, mock, and CD44v3 overexpressing HESCs. (B) Quantification of the outgrowth area. The mean value of the control group was set to 1. *p < 0.05

The effects of knockdown and overexpression of CD44v3 on primary HESCs were examined using western blot analysis (Fig. 4A–D, *p* < 0.05). The knockdown/overexpression of CD44v3 did not affect the rate of cell migration (Fig. 4E–F). Cell proliferation (Fig. 4G) and expression of PRL and IGFBP1 (Fig. 4H–K, *p* < 0.05) were significantly reduced following the knockdown of CD44v3. In addition, cell proliferation (Fig. 4G, *p* < 0.05) and expression of PRL and IGFBP1 (Fig. 4H–K, *p* < 0.05) were significantly increased following the overexpression of CD44v3. We further extended our observations to a heterologous coculture system of embryo implantation, which mimics the process of trophoblast invasion in endometrial stroma during in vivo implantation. After 24 h of coculture, the CD44v3-knockdown HESCs displayed a marked deficiency in supporting trophoblast outgrowth, whereas CD44v3-overexpressing HESCs significantly promoted the outgrowth (Fig. 5, *p* < 0.05).

## Discussion

RIF, with impaired endometrial receptivity, remains a significant challenge in assisted reproductive technology. The rates of implantation, even for perfectly healthy blastocysts, are still dependent on adequate decidualization of the uterus, allowing it to be receptive to the embryo [15, 16]. In the present study, we demonstrate, for the first time, that the expression levels of CD44v3 are decreased in the mid-secretory phase of the endometrial stroma in women with RIF, which affects endometrial receptivity via the inhibition of proliferation and decidualization of endometrial stromal cells.

CD44 family members are widely expressed transmembrane glycoproteins that participate in various cellular processes, including regulation of cell proliferation, division, migration, and adhesion [9]. Moreover, CD44 glycoproteins are critical mediators of tumorigenesis, endometriosis, and embryo epithelial interaction [17]. However, the functional role of CD44 family members in regulating endometrial receptivity in RIF patients remains unknown. We found that during the mid-secretory phase, when embryo implantation occurs, both CD44s and CD44v3 were widely expressed in glandular epithelial, luminal epithelial, and stromal cells. Although the expression of CD44v6 was previously reported to be restricted to the epithelial glands during the secretory phase, our results show that CD44v6 is expressed in both the endometrial epithelium and stroma during the mid-secretory phase.

Next, we explored the expression of the CD44 family members in the RIF and control groups. No differences were found in endometrial CD44s and CD44v6 expression between the two groups. For the first time, the present study confirms that the expression of CD44v3 is significantly decreased in the mid-secretory endometrial stromal cells of patients with RIF than in those with secondary infertility, but with no statistically significant changes in the expression in endometrial epithelial cells. Furthermore, ROC analysis revealed high sensitivity and specificity to discriminate the implantation status based on the expression levels of CD44v3 in endometrial tissues. To further investigate the relationship between CD44v3 and endometrial receptivity, we performed immunohistochemical and western blot analyses to determine the expression of CD44v3 during the menstrual cycle in normal women. The expression of CD44v3 was found to be significantly higher during the mid-secretory phase of human endometrial stromal cells than during the proliferation phase. We also aimed to identify the possible inducers of the CD44v3 expression during the mid-secretory phase. It is reported that immune cell subtypes, including uterine natural killer (uNK) cells, T cells, and B cells change dynamically in local endometrium during the menstrual cycle [18], and an implantation window accompanied the accumulation of uNK cells infiltration in the endometrium [19]. The expression of CD44v3 inform was reported in immune cells, such as T and B cells [20]; our results show that CD44v3 barely colocalizes with CD45 (leukocyte common antigen) both in RIF and controls (Supplemental Fig. 2); thus we believe that the quantitative change in CD44v3-positive cells is irrelevant to immune cell infiltration during the secretory phase. During the menstrual cycle, ovarian steroid hormones interact to prepare the endometrium for implantation. Accordingly, estradiol has been reported to increase the expression of CD44 [21, 22]. Our results show that CD44v3 is induced by E_2_ and P_4_ only in primary HESCs, instead of HEECs. Collectively, these findings suggest that the decreased expression of CD44v3 in the mid-secretory endometrial stromal cells may be a vital factor in RIF.

It has been established that endometrial stromal decidualization is crucial for successful embryo implantation. To create a receptive environment for blastocysts, endometrial stromal cells undergo proliferation during the estrogen-dependent phase of the menstrual cycle, followed by differentiation into decidual secretory cells under the influence of progesterone and estrogen [7, 23]. CD44v3 (CD44v3–v10), containing CD44 variable exons 3–10, has been confirmed to play an important role in the proliferation of various types of cancer cells [24]. In the present study, we found that cell proliferation was decreased following the knockdown of CD44v3, and the proliferation rate increased following the overexpression of CD44v3 in both T-HESCs and HESCs, consistent with the results of a recent study demonstrating that CD44 promotes the proliferation and growth of decidual stromal cells (DSCs) by binding to high molecular weight HA [11]. Because appropriate proliferation of endometrial stromal cells is necessary for decidualization, attenuation of proliferation inhibited decidualization. We further investigated the effect of CD44v3 on decidualization of stromal cells. In the in vitro decidualization assay, we found that the expression of PRL and IGFBP1, the two decidualization markers, was notably decreased following the knockdown of CD44v3 and was significantly increased following the overexpression of CD44v3 in HESCs. Furthermore, the CD44v3-knockdown HESCs displayed a marked deficiency in supporting trophoblast outgrowth, whereas the CD44v3-overexpressing HESCs promoted trophoblast outgrowth. These results indicate that in the mid-secretory endometrium, decreased expression of CD44v3 can inhibit the proliferation and decidualization of HESCs. In Ishikawa cells, which possess apical adhesiveness and similar characteristics as endometrial epithelial cells, there was not much difference in cell migration, proliferation, and adhesiveness capabilities after the overexpression/knockdown of CD44v3 (Supplemental Fig. 3). Overall, we show that the decreased expression of CD44v3 inhibited the proliferation and decidualization of stromal cells in women with RIF, which might contribute to impaired endometrial receptivity.

## Conclusions

We demonstrate, for the first time, that the expression of CD44v3 is higher during the mid-secretory phase of human endometrial stromal cells than it is during the proliferation phase, and shows a significant decline in the endometrial stroma of women with RIF. This indicates that low expression of CD44v3 might suppress proliferation and decidualization of stromal cells, further impairing endometrial receptivity in women with RIF.

## Supplementary Information

**Additional file1: Supplemental figure 1.** CD44v6 showed three different expression patterns in endometrial tissues Immunohistochemical staining for assessing the expression of CD44v6 in the control and RIF groups (*n* = 15 per group). Bar = 50 µm. **Supplemental figure 2.** Staining of CD44v3 and CD45 in mid-secretory phase endometrial specimens from RIF and control (Ctrl) women. Immunofluorescence staining for assessing the colocalization of CD44v3 and CD45 in the control and RIF groups (*n* = 4 per group). Bar = 20 µm. **Supplemental figure 3.** Effects of CD44v3 knockdown/overexpression on the cell migration, proliferation, and adhesiveness capabilities of Ishikawa cells. (A) Wound healing analysis and (B) semiquantitative analysis of wound closure in Ishikawa cells. (C) Cell proliferation after the knockdown and overexpression of CD44v3 in Ishikawa cells. (D and E) In the cell adhesion assay, the number of attached JAR cells was calculated and expressed as a fold change in the negative group. *p < 0.05.

**Additional file2: Table SI** Primers used in this study.

## Data Availability

The datasets in this study are available from the corresponding authors on reasonable request.

## Abbreviations

IVF: in vitro Fertilization
RIF: Recurrent implantation failure
T-HESCs: Immortalized human endometrial stromal cells
CD44s: Standard form of CD44
CD44v: CD44 variants
HEECs: Human endometrial epithelial cells
HESCs: Human endometrial stromal cells
