# Supplementary figures and images for: Decreased CD44v3 expression impairs endometrial stromal cell proliferation and decidualization in women with recurrent implantation failure
Source: Reprod Biol Endocrinol. 2022 Dec 16;20:170. doi: 10.1186/s12958-022-01042-w (PMC9756673; doi:10.1186/s12958-022-01042-w)

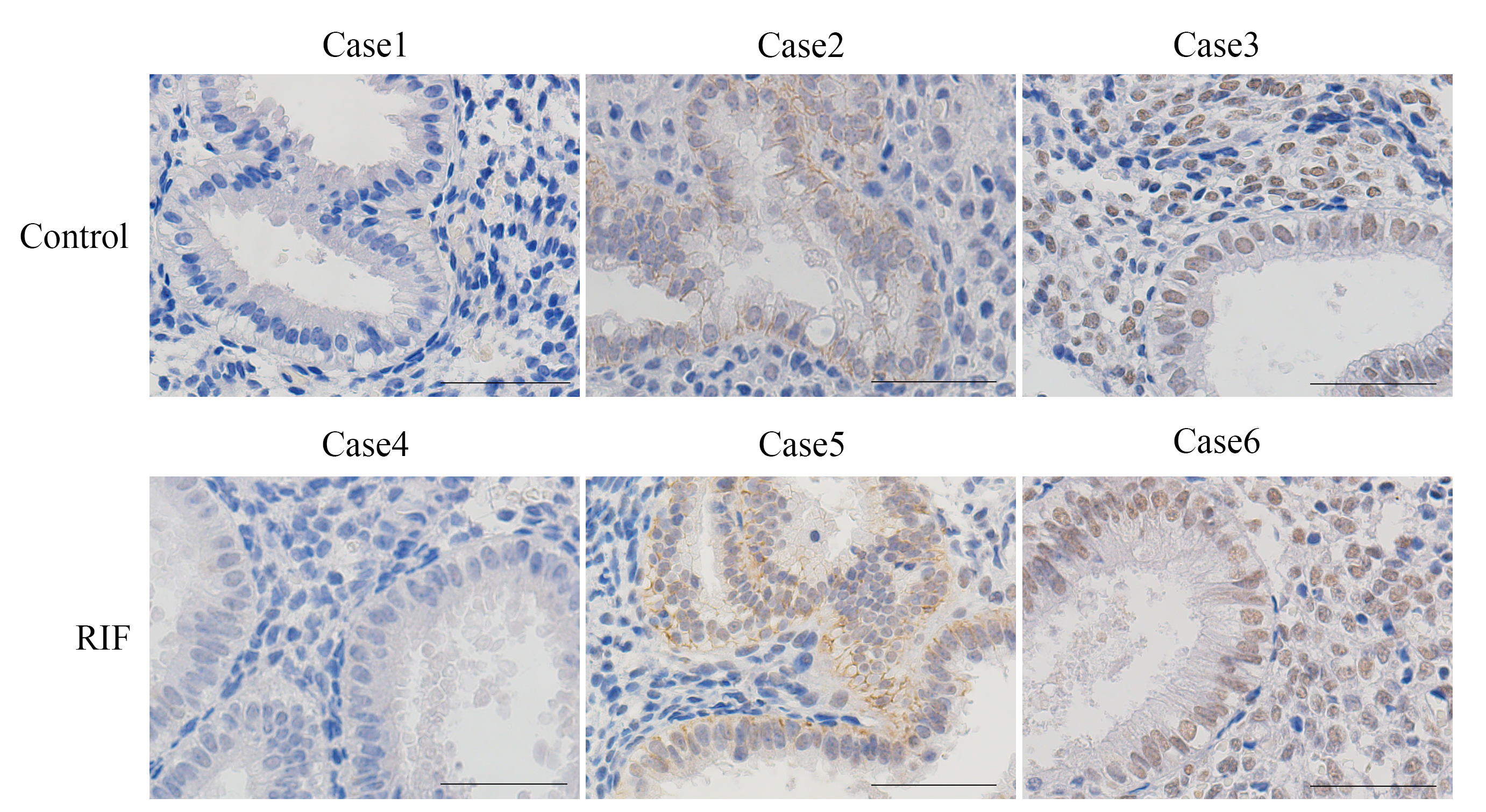

Supplement: Supplementary file 1 — Additional file1: Supplemental figure 1. CD44v6 showed three different expression patterns in endometrial tissues Immunohistochemical staining for assessing the expression of CD44v6 in the control and RIF groups (n = 15 per group). Bar = 50 µm. Supplemental figure 2. Staining of CD44v3 and CD45 in mid-secretory phase endometrial specimens from RIF and control (Ctrl) women. Immunofluorescence staining for assessing the colocalization of CD44v3 and CD45 in the control and RIF groups (n = 4 per group). Bar = 20 µm. Supplemental figure 3. Effects of CD44v3 knockdown/overexpression on the cell migration, proliferation, and adhesiveness capabilities of Ishikawa cells. (A) Wound healing analysis and (B) semiquantitative analysis of wound closure in Ishikawa cells. (C) Cell proliferation after the knockdown and overexpression of CD44v3 in Ishikawa cells. (D and E) In the cell adhesion assay, the number of attached JAR cells was calculated and expressed as a fold change in the negative group. *p < 0.05. [file 12958_2022_1042_MOESM1_ESM.zip › sFig.1.tif]

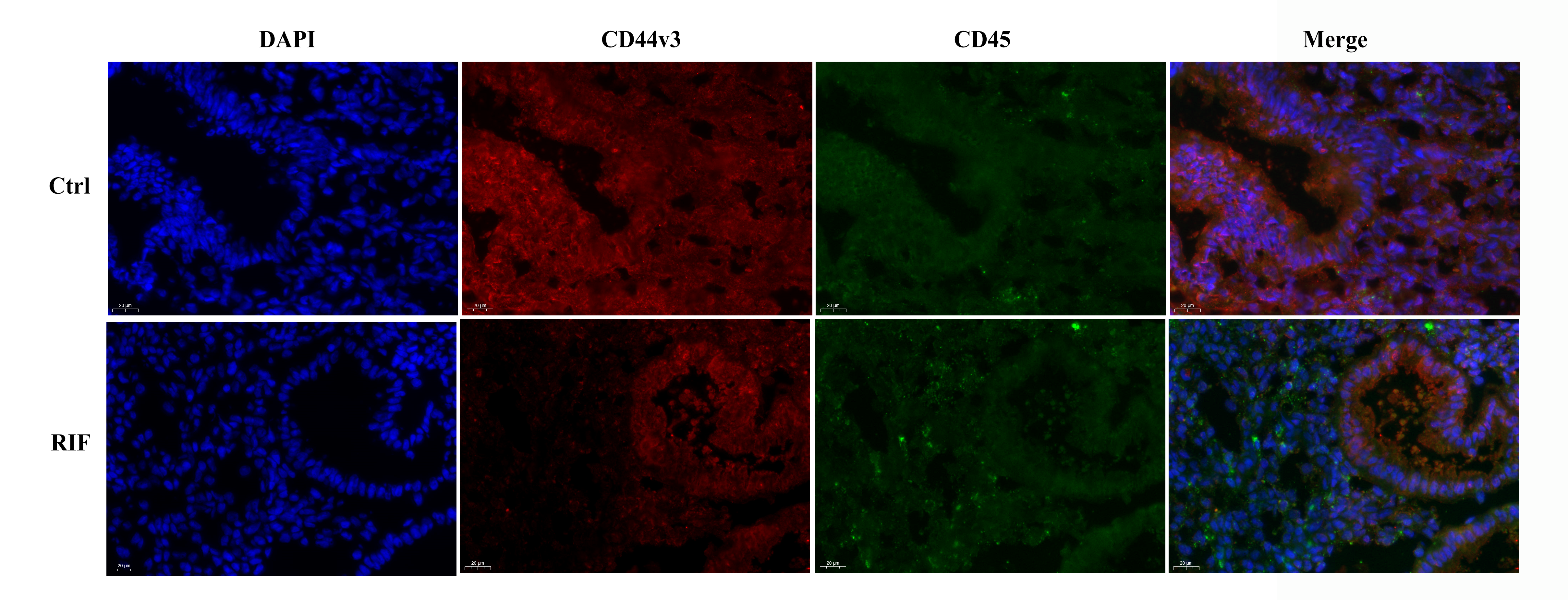

Supplement: Supplementary file 1 — Additional file1: Supplemental figure 1. CD44v6 showed three different expression patterns in endometrial tissues Immunohistochemical staining for assessing the expression of CD44v6 in the control and RIF groups (n = 15 per group). Bar = 50 µm. Supplemental figure 2. Staining of CD44v3 and CD45 in mid-secretory phase endometrial specimens from RIF and control (Ctrl) women. Immunofluorescence staining for assessing the colocalization of CD44v3 and CD45 in the control and RIF groups (n = 4 per group). Bar = 20 µm. Supplemental figure 3. Effects of CD44v3 knockdown/overexpression on the cell migration, proliferation, and adhesiveness capabilities of Ishikawa cells. (A) Wound healing analysis and (B) semiquantitative analysis of wound closure in Ishikawa cells. (C) Cell proliferation after the knockdown and overexpression of CD44v3 in Ishikawa cells. (D and E) In the cell adhesion assay, the number of attached JAR cells was calculated and expressed as a fold change in the negative group. *p < 0.05. [file 12958_2022_1042_MOESM1_ESM.zip › sFig.2.tif]

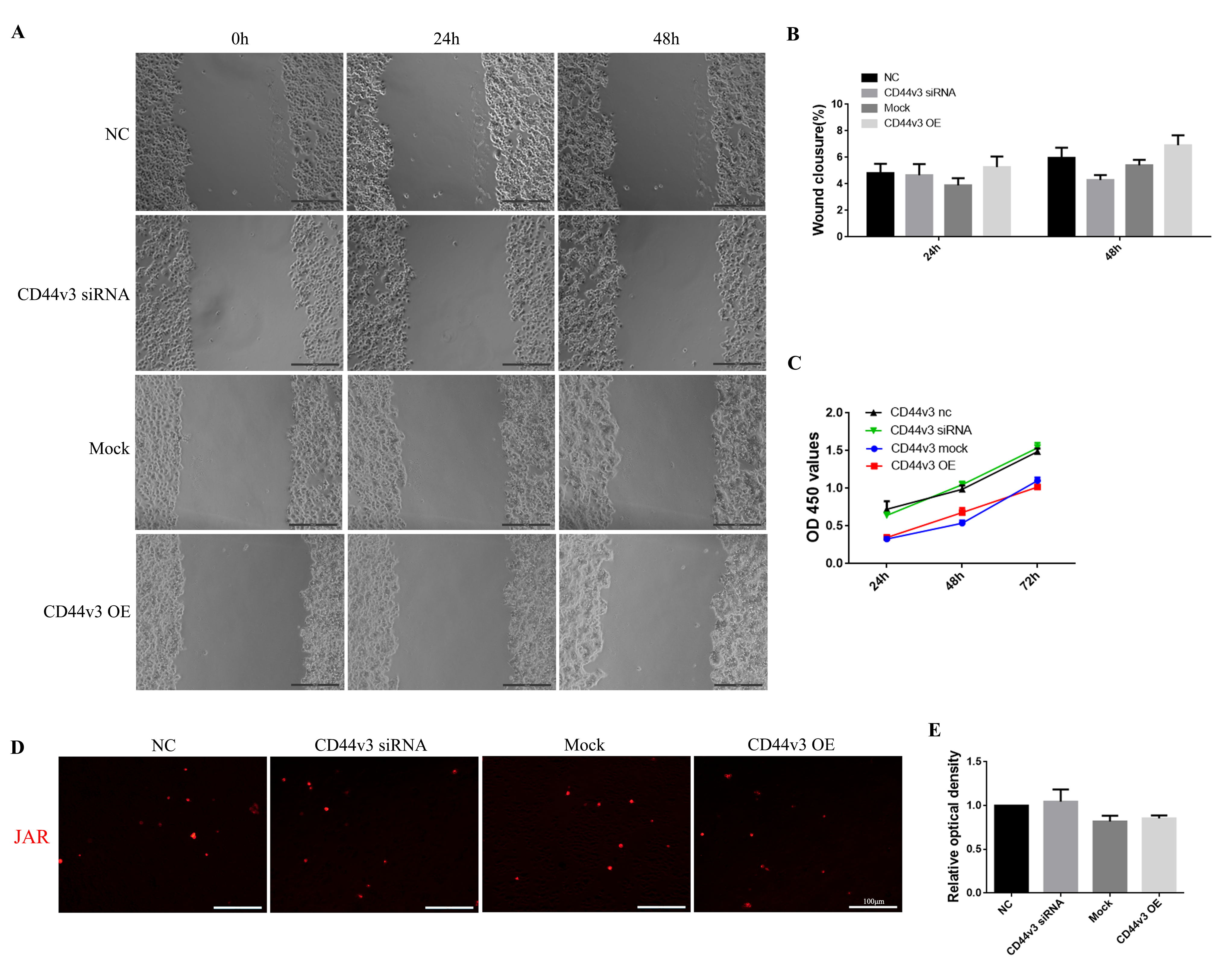

Supplement: Supplementary file 1 — Additional file1: Supplemental figure 1. CD44v6 showed three different expression patterns in endometrial tissues Immunohistochemical staining for assessing the expression of CD44v6 in the control and RIF groups (n = 15 per group). Bar = 50 µm. Supplemental figure 2. Staining of CD44v3 and CD45 in mid-secretory phase endometrial specimens from RIF and control (Ctrl) women. Immunofluorescence staining for assessing the colocalization of CD44v3 and CD45 in the control and RIF groups (n = 4 per group). Bar = 20 µm. Supplemental figure 3. Effects of CD44v3 knockdown/overexpression on the cell migration, proliferation, and adhesiveness capabilities of Ishikawa cells. (A) Wound healing analysis and (B) semiquantitative analysis of wound closure in Ishikawa cells. (C) Cell proliferation after the knockdown and overexpression of CD44v3 in Ishikawa cells. (D and E) In the cell adhesion assay, the number of attached JAR cells was calculated and expressed as a fold change in the negative group. *p < 0.05. [file 12958_2022_1042_MOESM1_ESM.zip › sFig.3.tif]
