# Supplementary material for: Decreased CD44v3 expression impairs endometrial stromal cell proliferation and decidualization in women with recurrent implantation failure
Source: Reprod Biol Endocrinol. 2022 Dec 16;20:170. doi: 10.1186/s12958-022-01042-w (PMC9756673; doi:10.1186/s12958-022-01042-w)
Supplement: Supplementary file 2 — Additional file2: Table SI Primers used in this study. [file 12958_2022_1042_MOESM2_ESM.docx]

Table SI Primers used in this study.

| Gene | Sequence (5’-3’) |
| --- | --- |
| CD44-F | CTGCCGCTTTGCAGGTGTA |
| CD44-R | CATTGTGGGCAAGGTGCTATT |
| CD44v3-F | TCCCTGCTACCAATATGGAC |
| CD44v3-R | CTCTGCTGCGTTGTCATTGA |
| CD44v6-F  CD44v6-R | GGCAACAGATGGCATGAGGG  AGTGGTATGGGACCCCCCACTGGGG |
| PRL-F | AGGAGCAAGCCCAACAGATG |
| PRL-R | TACTTCCGTGACCAGATGATACAG |
| IGFBP1-F | CCCAGAGAGCACGGAGATAAC |
| IGFBP1-R | GGTGACATGGAGAGCCTTCG |
| GAPDH-F | TGACTTCAACAGCGACACCCA |
| GAPDH-R | CACCCTGTTGCTGTAGCCAAA |
